# Coupling Energy Capture and Storage – Endeavoring to make a solar battery

**DOI:** 10.1038/s41598-018-30728-8

**Published:** 2018-08-24

**Authors:** Yukti Arora, Shateesh Battu, Santosh Haram, Deepa Khushalani

**Affiliations:** 1 Department of Chemical Sciences, Tata Institute of Fundamental Research, Mumbai, 400005 India; 2 Department of Chemistry, Savitribai Phule Pune University, Pune, 411007 India

## Abstract

Storage of solar radiation is currently accomplished by coupling two separate devices, one that captures and converts the energy into an electrical impulse (a photovoltaic cell) and another that stores this electrical output (a battery or a supercapacitor electrochemical cell). This configuration however has several challenges that stem from a complex coupled-device architecture and multiple interfaces through which charge transfer has to occur. As such presented here is a scheme whereby solar energy capture and storage have been coupled using a single bi-functional material. Two electroactive semiconductors BiVO_4_ (n-type) and Co_3_O_4_ (p-type) have been separately evaluated for their energy storage capability in the presence and absence of visible radiation. Each of these have the capability to function as a light harvester and also they have faradaic capability. An unprecedented aspect has been observed in that upon photo-illumination of either of these semiconductors, *in situ* charge carriers being generated play a pivotal role in perturbing the electroactivity of the redox species such that the majority charge carriers, *viz*. electrons in BiVO_4_ and holes in Co_3_O_4_, influence the redox response in a disproportionate manner. More importantly, there is an enhancement of *ca*. 30% in the discharge capacity of BiVO_4_ in the presence of light and this directly provides a unique route to augment charge storage during illumination.

## Introduction

Devising energy schemes that merge energy capture with energy storage have gained momentum over the last few years^1–3^. The impetus stems from utilizing solar radiation efficiently in terms of not only capturing it but also viably storing it in the form of either solar fuels or as electrical storage. The latter technology involving electrical storage is still emerging especially in terms of evolving the conceptual idea of *directly* storing solar radiation as opposed to forming devices that consist of independent batteries/supercapacitors that are separately coupled with solar cells. For viable energy storage to occur, effective high energy electrons are used either to charge a capacitive layer or they assist in effecting a faradaic (redox) process. Generating these high energy carriers using a photo-assisted process is now being exploited using technologies involving DSSC (dye sensitized solar cells), photoelectrochemical or photochemical assisted production of high energy electrons and these are subsequently being interfaced with energy storage electrodes^1–4^. Hence, it can be considered that the storage systems are composed of two kinds of materials: light harvesting and a storage component. Light harvesting component consists of materials that are capable of absorbing light and generating extractable charge carriers, while, energy storage component consists of materials that can trap the charges and store them during periods of illumination, and subsequently release them under discharge conditions.

Over the last few years, semiconductors such as WO_3_^5^, TiO_2_^6^, Ni(OH)_2_^7^, MoO_3_^8^, Co_3_O_4_^9^, and V_2_O_5_^10^ have been extensively studied as the active electrode materials for energy storage. Moreover, there are a number of reports where the aforementioned energy storage components have been interfaced with light absorbers to form a coupled device. WO_3_/TiO_2_ and Ni(OH)_2_/TiO_2_ are two of the most widely studied hybrid energy storage systems^4,11,12^, where in the former case, photo generated electrons are usually stored in the form of tungsten bronze (Na_x_WO_3_), while in the latter case photo generated holes are stored in the form of chemical reaction where conversion of Ni^2+^ to Ni^3+^ takes place. TiO_2_ (band gap 3.2 eV) is the most frequently used light harvesting material and therefore in most of the hybrid photoelectrodes UV light is used for photocharging^13^. However, to exploit the solar spectrum more efficiently, various approaches such as coupling TiO_2_ with dyes^14^ or transition metal (Pt^13^, Au, Ag^8^) nanoparticles or incorporating dopants have been adopted to design visible light responsive components but unfortunately the devices show poor efficiencies mainly owing to multiple interfaces being involved. Recently Gimenez *et al*. have shown solar energy storage in a photocapacitive device coupled with BiVO_4_ where this moiety serves the purpose of a light absorber and it has been done in unison with PbO_x_ as the capacitive layer^3^. From already existing work, what is being learnt is that configuring the two components in a single device to harness and store solar energy is a complex process owing to stringent requirements of a variety of parameters such as semiconductor band gap, its alignment with the electrode/electrolyte, charge transport kinetics, energy conversion efficiency, and material stability.

We have presented an alternate approach for coupling energy capture and storage in which the focus has been to create a strategy that minimizes interfaces and so in principle can lead to better performance and charge transport efficiency. This we believe is a seminal report that studies the impact of visible light on the semiconductor material which is *also* an electroactive material that stores energy electrochemically. This work allows the removal of multiple interfaces by using bi-functional materials and the results show that *in situ* charge generation and storage can be made viable. Towards this aim, two semi-conductors have been evaluated exclusively for storage properties in the direct presence and absence of visible light irradiation. Specifically, one dimensional structures of BiVO_4_ (n-type semi-conductor) and Co_3_O_4_ (p-type semi-conductor) have been synthesized, structurally characterized, and their individual electrochemical behavior has been evaluated. Subsequently, their redox behavior in the presence and absence of light has been compared in order to show that it is feasible for an electroactive component to be also photoactive in a single energy storage device.

BiVO_4_ is an n-type semiconductor with a monoclinic scheelite structure, and is widely being studied as a photoanode for solar water oxidation owing to its small band gap (*ca*. 2.3 eV), it utilizes a significant portion of solar spectrum and its valence band edge position is appropriate for water oxidation^15^. The second semiconductor that has been evaluated is Co_3_O_4_. This is a p-type semiconductor, crystalizes in three dimensional normal spinel structure and has gained considerable attention as water oxidation/oxygen evolution catalyst^16^. It should be highlighted, that individually each of these semiconductors have been evaluated in pure and hybrid based supercapacitor applications as well. Both the semiconductors *viz*. BiVO_4_ and Co_3_O_4_ have a 3-D crystal structure unlike most of the conventional layered materials employed for energy storage. In the last few years, BiVO_4_ has shown to be a good electroactive material especially in conjunction with CNTs^17^ and with nanostructured MoS_2_^18^. BiVO_4_ undergoes reversible faradaic change (Bi^3+^ ↔ Bi^0^) on ramping the voltage and stores energy electrochemically. Also, Co_3_O_4_ has been employed as an attractive energy storage material owing to its low cost, impressive redox activity, and most importantly, high theoretical specific capacitance (890 mA hg^−1^)^19^. However, it should be noted that in none of these reports were the behavior of these compositions evaluated while being impinged with visible irradiation.

## Results and Discussion

**Figure 1:**
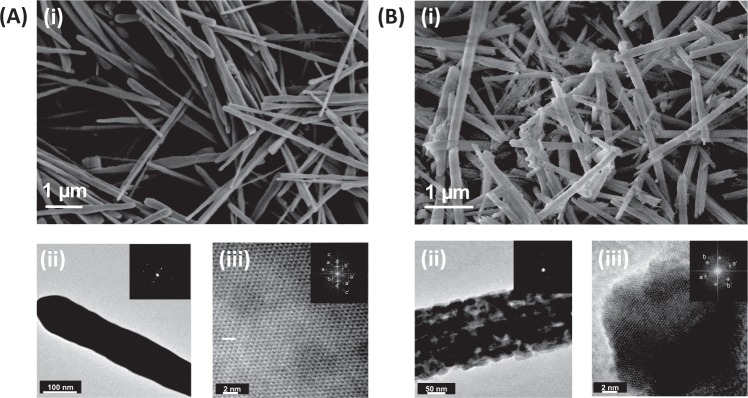
Electron micrographs of BiVO_4_ and Co_3_O_4_ rods (**A**) i: SEM image of solvothermally synthesized BiVO_4_ nanorods with diameter ~113 nm and length ~6 µm, ii: TEM image showing uneven surface of these rods, inset shows its SAED pattern acquired from a single particle indicating high crystallinity as depicted by the bright spots, and iii: HRTEM image of BiVO_4_ nanorods showing a defined lattice fringe, inset showing FFT image where (aa′), (bb′), and (cc′) corresponds to (112), (101/001) and (301) plane, respectively; (**B**) i: SEM image of Co_3_O_4_ rod like structures with diameter ~143 nm and length ~6 µm, inset shows its SAED pattern with well-defined diffraction rings depicting polycrystallinity of Co_3_O_4_, ii: TEM image showing 1-D structure of Co_3_O_4_ formed by agglomeration of nanocrystals, and iii: HRTEM image of Co_3_O_4_ rods, inset showing the FFT image with (aa′) and (bb′) corresponding to (112) and (311) planes, respectively.

A highly modified synthesis protocol yielded monodispersed rod-like structures of monoclinic phase of BiVO_4_ using a solvothermal synthesis route. Figure 1(A,i) shows SEM image of BiVO_4_, the morphology consists of rod like particles, which are highly crystalline as confirmed by the selected area electron diffraction (SAED) pattern acquired from a single rod, inset Fig. 1(A,ii). Average length and diameter of BiVO_4_ rods was determined to be 6.62 ± 0.62 µm and 113 ± 40 nm, respectively. HRTEM image of BiVO_4_ shows regular ordering with an FFT image (inset of Fig. 1(A,iii)) showing a variety of planes being present suggesting polycrystallinity. BiVO_4_ is an intrinsically n-type semiconductor^20^, with a band gap of *ca*. 2.3 eV. Diffuse reflectance spectrum (DRS), Supplementary Fig. 1, shows that BiVO_4_ absorbs a significant portion of the visible light and the band edge was determined to be at 2.23 eV which corroborates well with literature values^21^. Using N_2_ adsorption desorption measurements, BET surface area of these BiVO_4_ rod like structures was determined to be *ca*. 13 m^2^/g.

Analogously, Co_3_O_4_ was also synthesized in a rod-like structure *via* facile hydrothermal synthesis followed by calcination. It can be seen in the SEM, Fig. 1(B,i) that the final product after calcination consists of uniform rod-like structures with length in the range of 6.35 ± 0.45 µm and diameter in the range of 143 ± 27 nm. TEM image reveals that these 1-D Co_3_O_4_ structures consist of aggregated nanocrystals with diameter of 15.5 ± 2.4 nm, Fig. 1(B,i). The lattice fringes in the HRTEM image indicate that Co_3_O_4_ is well ordered, Fig. 1(B,iii), which is further supported by the SAED pattern (acquired from a single rod) that shows material actually is polycrystalline as well-defined diffraction rings were obtained, inset Fig. 1(B,ii). DRS spectrum shows Co_3_O_4_ absorbs significantly in the near UV as well as visible region, and its band gap is known to be 1.6 eV, Supplementary Fig. 1. N_2_ adsorption desorption measurements provided a BET surface area for these Co_3_O_4_ rods to be *ca*. 39.4 m^2^/g.

**Figure 2:**
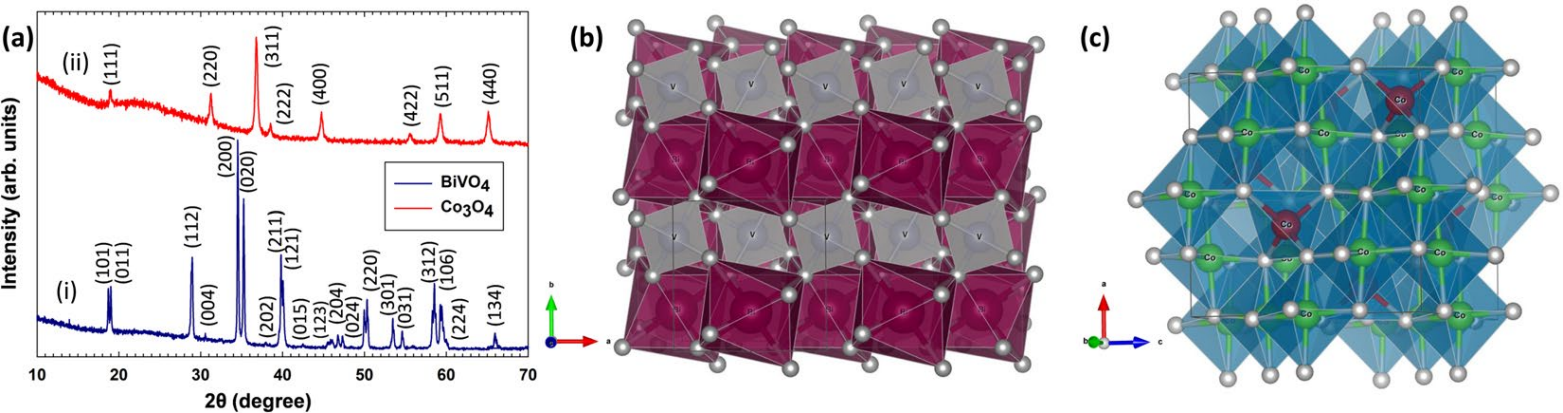
(**a**) XRD patterns of (i) BiVO_4_ rods with preferential orientation along (200) plane that can be indexed to JCPDS no. 04-010-5713, and (ii) Co_3_O_4_ rod like structures, where all the peaks can be indexed to JCPDS no. 43–1003, (**b**) represents polyhedral structure of BiVO_4_, where magenta colored polyhedra are BiO_8_ units whereas grey colored polyhedra are VO_4_ units and the two sets of polyhedra form layered structure with alternating layers, and (**c**) represents polyhedral structure of Co_3_O_4_ where red atoms are Co^2+^ ions occupying tetrahedral interstices whereas green atoms are Co^3+^ ions occupying octahedral interstices. Grey balls in both the structures are oxygen atoms.

The purity and crystallinity of BiVO_4_ as-synthesized material was analyzed by high resolution X-ray diffraction. The diffraction pattern can be indexed to JCPDS database (04-010-5713), corresponding to monoclinic scheelite structure, Fig. 2(a,i). In the scheelite structure, each V ion is coordinated to four O atoms in a tetrahedral site while each Bi ion is coordinated by eight O atoms each from a different VO_4_ tetrahedral unit^22^. Therefore, basic structural unit of monoclinic BiVO_4_ constitutes of VO_4_ tetrahedron and BiO_8_ dodecahedron. Each oxygen atom of VO_4_ tetrahedron unit is coordinated to a different Bi atom. There are four types of Bi-O bonds ranging from 2.354 to 2.628 Å and two types of V-O bonds are 1.692 and 1.767 Å. The polyhedral representation of this structure provides an interesting insight into the material where it can be considered to consist of a *layered* structure of alternating layers of VO_4_ tetrahedron (grey in color) and BiO_8_ dodecahedron (magenta in color), Fig. 2b. Importantly, Zou *et al*. have reported for these two different types of polyhedral, there exists a strong hybridization between V and O atoms, forming covalent bonding, V-O dipole, in the VO_4_ tetrahedra; however, there exists a relatively weak hybridization between Bi and O atoms, resulting in the ionic bonding, Bi-O dipole, in BiO_8_ dodecahedra^23^. This is important as it lends to the idea that in principle Bi^3+^ can more easily diffuse out/in from the framework as opposed to the V^5+^.

Analogously, Co_3_O_4_ was also synthesized using a hydrothermal protocol and Fig. 2(a,ii) shows the representative PXRD pattern. All the peaks in the X-Ray diffractogram of Co_3_O_4_ can be indexed to a cubic spinel structure of Co_3_O_4_ with lattice constant, a = 8.08 Å (JCPDS No. 43–1003, space group: *Fd*3*m*). In Co_3_O_4_ structure, cobalt ions exist in two different oxidation states *i*.*e*. Co^2+^ and Co^3+^. Co^2+^ ions occupy tetrahedral interstices, represented in red color and Co^3+^ ions occupy octahedral interstices, represented in green color in Fig. 2c. All Co(II)-O bond lengths are 1.948 Å and all Co(III)-O bond lengths are 1.935 Å. Shorter Co(III)-O bond length is the result of a stronger interaction between Co^3+^ and O, this would in turn affect the oxidation/reduction potentials of the two different cobalt ions.

### Electrochemical Measurements

**Figure 3:**
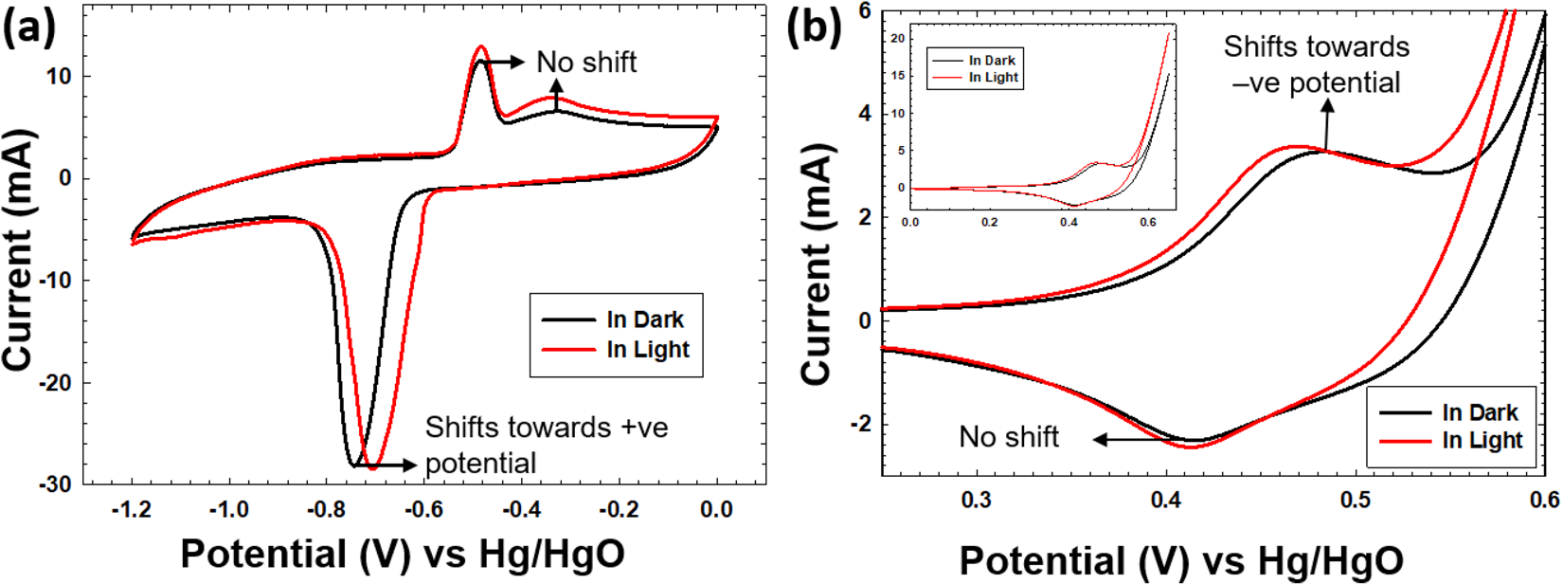
(**a**) CV of BiVO_4_ in the presence and absence of light, light causes augmentation in the area under oxidation/reduction peaks and shifts the reduction peak towards positive potential, and (**b**) CV of Co_3_O_4_ in the presence and absence of light, light again affects the CV of Co_3_O_4_ in two ways: augmentation in the area under oxidation/reduction peak and shift in the oxidation peak potential towards negative potential. BiVO_4_ (n-type semiconductor) and Co_3_O_4_ (p-type semiconductor) behave symmetrically opposite in the presence of light. All the CV curves have been acquired at a scan rate of 20 mVs^−1^.

The electrochemical behavior of BiVO_4_ was evaluated using three electrode system by performing CV under different scan rates ranging from 5 to 100 mVs^−1^. However, for sake of brevity, the data shown here is for 20 mVs^−1^ under the potential window 0.0 V to −1.2 V, Fig. 3a (black curve). Extraneous stray light was avoided by carrying out the experiment in a closed black box. Under dark conditions, the conventional BiVO_4_ CV curve is obtained. Faradaic peaks in the CV curve can be attributed to quasi reversible redox process, Bi^3+^ ↔ Bi metal. It shows single cathodic peak at −0.75 V which can be ascribed to single step reduction of Bi^3+^ to Bi° and the two anodic peaks at −0.50 V and −0.38 V correspond to the two step oxidation of Bi^0^ to Bi^+^ and Bi^+^ to Bi^3+^, respectively. The redox peaks were reproducible at higher scan rates, however the oxidation peaks shift towards positive potentials and reduction peak shifts towards negative potential which is mainly ascribed to the internal resistance^18^, Supplementary Fig. 2. Augmentation in the current response was observed at higher scan rates which indicates faster interfacial redox kinetics. As mentioned above, the bonding between Bi and O is relatively weaker when compared to the interaction between V and O, as a consequence the faradaic behavior of solely Bi ion is observed in the cyclic voltammogram. It is anticipated that BiVO_4_ structure lends itself to a feasible diffusion of Bi ion in and out of the lattice when the material is subjected to charging and discharging, respectively and the vanadate units are potentially only contributing to the pseudocapacitive aspects of the CV curve. As previously reported by Khan *et al*.^17^ and Arora *et al*.^18^ BiVO_4_ has shown decent cycling stability considering it bears a 3-D crystal structure unlike conventional layered battery materials. Supplementary Fig. 3a,b show the SEM images of the working electrode before and after cyclic voltammetry, in the former image BiVO_4_ rods with a flake like morphology which correspond to the additives *viz*. polyvinylidene difluoride (PVDF, binder) and activated carbon are visible. However, in the SEM image after 20 CV cycles, BiVO_4_ rods are visible and the ill-defined morphology is now a combination of Bi_2_O_3_, NaVO_3_, V_2_O_7_, and the additives. The peaks in the diffraction pattern of the working electrode after 20 CV cycles can be indexed to Bi_2_O_3_, NaVO_3_ and V_2_O_7_ which reveal their formation during electrochemical measurements, Supplementary Fig. 3c. Bi ions diffuse out of the lattice while charging, perhaps it is the Na^+^ ions (of the electrolyte) that diffuse into the lattice to charge balance the system and this results in the formation of NaVO_3_. Ideally, if the electrolyte could be replaced with a Bi^3+^ ion based ionic liquid these issues could be circumvented. However even in the current scenario of using NaOH as the electrolyte, it should be noted that despite this irreversible damage, under the experimental setup enough BiVO_4_ is deposited such that cycling is still feasible for up to 70 cycles. Supplementary Fig. 4 shows the representative diagram highlighting how the working electrode gets modified with each CV cycle. The electrochemically active surface area (EASA) of the working electrodes was estimated to assess surface characteristic of the electrodes and its interaction with the electrolyte. EASA was estimated from the electric double layer capacitance of the electrode surface which was done by measuring the non-faradaic capacitive current under the potential window of −0.10 V to −0.14 V with scan rates ranging from 20 to 400 mVs^−1^ and as such we obtained EASA of the working electrodes as *ca*. 30 cm^2^. Supplementary Fig. 5a,b, detail the full analysis of the EASA measurements.

In order to investigate the electrochemical behavior of BiVO_4_ under light, a simple experiment was devised whereby BiVO_4_ was drop-cast onto graphite electrode, aforementioned 2 M NaOH was used as the electrolyte in a quartz flat cell, reference electrode was Hg/HgO, and the counter electrode was Pt foil. This entire assembly was then encased such that the CV could be recorded under ‘light’ conditions within the potential range 0.0 V to −1.2 V at 20 mVs^−1^. The light conditions involved irradiating the working electrode with a 100 W Tungsten lamp fitted with a 400 nm long pass filter. This was located at a working distance of 10 cm from the working electrode. Diagram of three electrode quartz cell where working electrode is being impinged with the visible light has been demonstrated in Supplementary Fig. 6.

**Figure 4:**
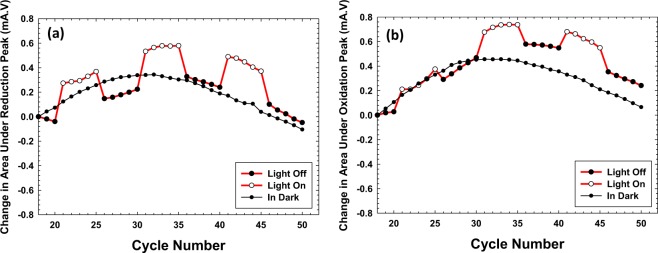
(**a** and **b**) Variation in the area under reduction and oxidation peak, respectively, as a function of cycle number on irradiating BiVO_4_ rods with visible light, black curve is the baseline acquired in dark and the non linear profile indicates instability of BiVO_4_ in NaOH whereas red curve corresponds to three sets of cycles showing how the area under reduction as well as oxidation peak gets augmented under ‘light on’ condition. Data (Y-axes) in both the figures (**a**) and (**b**) is normalized with respect to the area under the reduction and oxidation peak in cycle number 18, respectively.

**Figure 5:**
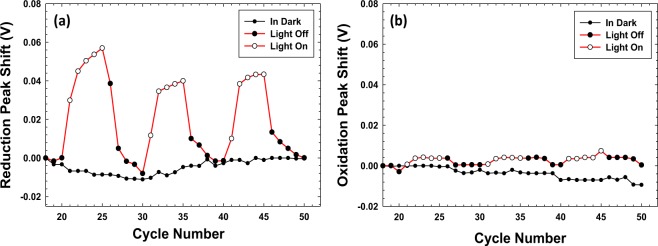
(**a**) Variation in the reduction peak potential at −0.75 V of BiVO_4_ as a function of cycle number, black curve is the baseline acquired in dark, red curve shows the shift in the reduction peak potential towards positive potential upon pulsating with visible light, an average shift of 49.9 mV has been recorded; (**b**) variation in the oxidation peak potential at −0.53 V of BiVO_4_ as a function of cycle number, black curve is again the baseline acquired in dark, where as the red curve shows a negligible oxidation peak shift on photoirradiation. Data (Y-axes) in both the figures (**a** and **b**) is normalized with respect to the reduction and oxidation peak potentials in cycle number 18, respectively.

Extraneous stray light was avoided under both dark and light conditions. As the working electrode was impinged with the visible light, the CV profile of BiVO_4_ was found to be intriguingly perturbed, Fig. 3a. Two aspects were clearly altered under ‘light’ conditions: an augmentation in the area under the cyclic voltammogram was observed and an even more intriguing observation was the shift in the reduction peak potential towards positive potential, however, no such deflection was observed in the oxidation peak potentials. In order to monitor this closely, the first 20 CV cycles were carried out in dark for stabilization and to avoid any influence due to the initial ‘activation process’^24,25^. Subsequently, *light-on* condition was activated on the 21^st^ cycle. The working electrode was illuminated for 5 cycles (shown with white circles) and the current was simultaneously measured, then the light was turned off for next 5 cycles (shown with dark circles), and then again the light was turned on for 5 cycles, Fig. 4a. This way BiVO_4_ (n-type semiconductor) was impinged with visible light in a pulsating mode and its redox behavior was carefully monitored. Figures 4 and 5 showcase the data whereby Fig. 4 shows the variation in the reduction/oxidation peak areas as a function of the cycle number (pulsed light on and off conditions) and Fig. 5 showcases the variation in the deflection of the reduction/oxidation peak potential again as a function of the cycle number. In both these figures, it should be noted that a baseline has been provided which is the change (if any) in the area or peak potential when the measurement (entire 50 cycles) have been done completely in the dark. Ideally, it should be anticipated that the area under the reduction/oxidation peaks should not vary as a function of the cycle number and hence this baseline should have a slope of zero, centered at 0 mA·V. However, it can be seen that this black curve in Fig. 4a (baseline in dark) appears to gradually increase and then decrease upon increasing the number of cycles. Currently, it has not been completely elucidated as to why the conductivity of the working electrode improves (an unusual observation compared to what has been reported in current literature^17^), however it can be stated that upon each cycle, the working electrode gets physically and chemically altered such that in the first few cycles the electrode-electrolyte interaction improves (positive slope of the baseline) and subsequently as mentioned above BiVO_4_ undergoes a slow degradation because of harsh electrolytic environment and therefore this obviously leads to decreased conductivity as noted by the downward slope of the baseline. This behavior has been reproducibly observed upon repeating the experiment with a fresh working electrode.

Despite this broad non-linear baseline, it is interesting to see a dramatic change in the area under the reduction/oxidation peaks upon pulsing with visible light. Three sets of cycles with white circles show how area under the reduction peak gets augmented under ‘light on’ conditions, Fig. 4a (red curve). Similar trend was also observed for the area under the oxidation peak, as this value also was augmented while the light was turned on, and subsequently decreased upon turning off the light, Fig. 4b (red curve). An average enhancement of *ca*. 288 mA.mV and 175 mA.mV have been recorded for area under the reduction and oxidation peaks, respectively. To measure the area under the oxidation peak, potential window −0.6 V to 0.0 V was chosen and area under the reduction peak was measured under the potential window −1.2 V to −0.6 V for both light and dark experiments. In principle, this observation can insinuate that a larger amount of BiVO_4_ is being probed electrochemically just by irradiating with light.

An even more interesting aspect of this study was an asymmetric behavior observed with respect to the shift in redox potentials. Figure 5a shows the change in the reduction peak potential (at −0.75 V) upon pulsing with light and the *lack of* change in the oxidation peak potential (at −0.50 V) upon pulsing with light, Fig. 5b. It is clearly observed that the reduction peak shifts towards positive potentials indicating that Bi^3+^ → Bi^0^ is now a more feasible reaction in the presence of light. The data has been normalized with respect to cycle number 18, reduction and oxidation peak potential values at cycle number 18 were −0.75 V and −0.53 V, respectively, which have been set to 0 V in order to get the absolute peak deflection values. On averaging the three sets of cycles in light and dark, a deflection of *ca*. 50 mV is seen for the reduction peak potential and a small shift of *ca*. 4 mV for the oxidation peak potential is recorded. From this, it can be inferred that the load on the external device to perform the reduction step can simply be reduced by illuminating the material with light. However, oxidation peak does not show any such significant deflection which implies that, in principle, the material gets charged at the same potential however, now it discharges at a higher potential. Also, on increasing the lamp power the extent of deflection in the reduction peak potential got increased, Supplementary Fig. 7.

Analogously, electrochemical behavior of Co_3_O_4_, which is a p-type semiconductor, was evaluated. Cyclic voltammetry studies were performed in the aforementioned three electrode assembly with operating potential window between 0.0 V and 0.65 V at varying scan rates in the range of 5 mVs^−1^ to 100 mVs^−1^, however for the sake of brevity, the data shown here is for 20 mVs^−1^, Fig. 3b (black curve). The pair of faradaic peaks in the CV curve of Co_3_O_4_, indicates that the capacitance of Co_3_O_4_ mainly stems from pseudocapacitance and not electric double layer capacitance^9^. Normally, under ideal conditions, two pairs of redox peaks have been reported for Co_3_O_4_ and they correspond to the conversion between different cobalt oxidation states according to the following redox processes:

$${{\rm{Co}}}_{3}{{\rm{O}}}_{4}+{{\rm{OH}}}^{-}+{{\rm{H}}}_{2}{\rm{O}}\leftrightarrow 3\,{\rm{CoOOH}}+{{\rm{e}}}^{-}$$

$${\rm{CoOOH}}+{{\rm{OH}}}^{-}\leftrightarrow {{\rm{CoO}}}_{2}+{{\rm{H}}}_{2}{\rm{O}}+{{\rm{e}}}^{-}$$

In our data, the pair of faradaic peaks in the CV of Co_3_O_4_ at 0.48 V and 0.41 V can be assigned to oxidation and reduction of Co ions, respectively. As can be seen, only one cathodic and anodic peak is observed in our case which could perhaps be due to simultaneous conversion of CoOOH (product of first oxidation process) to CoO_2_ and likewise reverse reduction process yields Co_3_O_4_. Hence, two anodic and two cathodic peaks are so close that they cannot be distinguished. It should be noted that similar behavior has also been observed in previous reports^9^. The increase in oxidation current at higher potentials is always attributed to the water oxidation process, inset Fig. 3b.

**Figure 6:**
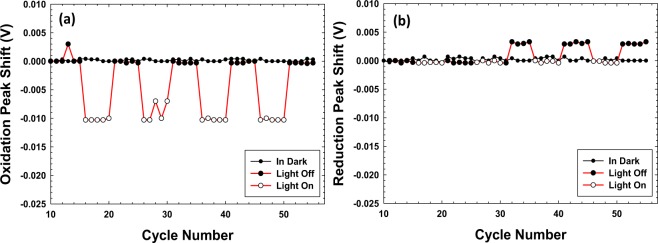
(**a**) Variation in the oxidation peak potential at 0.494 V of Co_3_O_4_ as a function of cycle number, black curve corresponds to the baseline acquired in dark, red curve shows the shift in the oxidation peak potential towards negative potential upon pulsating with visible light, an average shift of 11 mV has been recorded; (**b**) variation in the reduction peak potential at 0.40 V of Co_3_O_4_ as a function of cycle number, black curve is again the baseline acquired in dark, where as the red curve shows a negligible reduction peak shift on photoirradiation. Data (Y-axes) in both the figures (**a** and **b**) is normalized with respect to oxidation and reduction peak potential in cycle number 10, respectively.

On impinging Co_3_O_4_ with the visible light, its CV profile was also altered. On carefully analyzing the perturbation, it was observed that the area under the cyclic voltammogram was augmented just as in the aforementioned case of BiVO_4_, however a surprising observation was the shift in the oxidation peak potential towards less positive potential, Fig. 3b (red curve). No such deflection was observed in the reduction peak, except that the current value got increased. This was symmetrically opposite to what was observed for BiVO_4_. In case of Co_3_O_4_, first 15 cycles were carried out in dark to avoid the influence of activation process, and then light was turned on and off from cycle number 16 onwards for 5 cycles each. Again the variation of peak area under the oxidation/ reduction peak (Supplementary Fig. 8) and potential for the oxidation/reduction peak (Fig. 6) has been showcased. The data is normalized with respect to cycle number 10, where the oxidation and reduction peak potential values were 0.49 V and 0.40 V, respectively, which have been set to 0 V to obtain the absolute shifts in the peak potentials. Likewise change in area under the oxidation and reduction peak is with respect to cycle number 10 and the potential window used to measure the area under the oxidation and reduction peak is 0.4 V to 0.65 V and 0.38 V to 0.43 V, respectively. It can be observed that the peak area is augmented for both the faradaic reactions (Supplementary Fig. 8), however the absolute values for the enhancement in area under oxidation and reduction peaks averaged over four sets of cycles are *ca*. 40 mA.mV and *ca*. 13 mA.mV, respectively. The values are not comparable because as mentioned above the potential windows fixed to measure the areas are different. The black curve in Fig. 6a,b correspond to the baseline, where all 55 CV cycles were carried out in dark. The deflection in the oxidation peak potential as averaged over four sets of cycles in light and dark is *ca*. 11 mV, however a small shift of *ca*. 2.7 mV in the reduction peak potential was observed, Fig. 6. Baseline in dark has been acquired by carrying out 55 CV cycles in the same setup in dark using a fresh working electrode. Co_3_O_4_ in the dark has shown much better stability compared to BiVO_4_ in 2 M NaOH electrolyte. Moreover, now in this scenario of utilizing a p-type semiconductor, the oxidation peak was deflected towards a lower positive potentials indicating Co^3+^ → Co^4+^ oxidation was now a more feasible reaction while the change in the reduction potential was minimal. Overall, it should be mentioned that alteration in the CV in the presence of light for Co_3_O_4_ was not large but the changes highlighted are thoroughly reproducible. It can therefore be surmised that upon irradiation with visible light Co_3_O_4_, in principle, gets charged at lower potentials but discharges at the same potential.

Considering one of the main differences between BiVO_4_ and Co_3_O_4_ is that the former is an n-type semiconductor and the latter a p-type semi-conductor, this lent to the idea that perhaps, upon irradiation with light, the *in situ* charge carriers being generated played a pivotal role as to which of the faradaic process: reduction or oxidation was being enhanced. Moreover, as the variation in the CV curves was asymmetric (*i*.*e*. only the reduction peak voltage predominantly deflected for BiVO_4_ and the oxidation peak voltage for Co_3_O_4_), it seemed that perhaps the electrons and holes were each influencing the system in a disproportionate manner. From the data, it can be inferred that in an n-type semiconductor, the presence of extra excited electrons (upon impinging with light) appear to contribute significantly to enhance the reduction component of the faradaic process while for a p-type semiconductor the reverse occurs, which is now there is higher preponderance of excited holes and these appear to enhance the oxidation process. In BiVO_4_ (n-type semiconductor) excited electrons formed on photoirradiation are more mobile and can reach the surface faster than the holes and hence, we observe its effect in the form of facile reduction *i*.*e*. deflection in the reduction peak is significant compared to the deflection in the oxidation peak. On the other hand, Co_3_O_4_ being a p-type semiconductor has a higher preponderance of holes on its surface which makes the oxidation peak shift by a significant value towards the lower potentials and reduction peak is unaffected.

**Figure 7:**
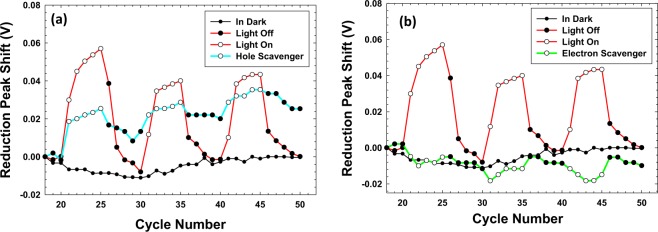
(**a**) Variation in the reduction potential (at −0.75 V) of BiVO_4_ in the presence of hole scavenger (Na_2_SO_3_) as a function of cycle number, a viable shift in the reduction peak potential is still observed on photoirradiation, (**b**) variation in the reduction potential of BiVO_4_ in the presence of electron scavenger (O_2_) as a function of cycle number, a complete suppression in the reduction peak shift upon irradiation is observed. Data (Y-axes) in both the figures (**a** and **b**) is normalized with respect to reduction peak potential in cycle number 18.

To gain further insight into this, scavengers (electron or hole) were added to the electrolyte and the same experiment was repeated *i*.*e*. BiVO_4_ working electrode, NaOH as the electrolyte and the system irradiated with light in pulsed mode (on for 5 CV cycles and off for 5 CV cycles). The aim of adding these scavengers was that they would nullify the extra concentration of the excited carriers and in principle it could be gauged if the asymmetric shifts in the CV curve could be reversed even during light irradiation. For the hole scavenger^26^, Na_2_SO_3_ was introduced into the system and analogously in a separate experiment, an electron scavenger^27^, O_2_ was introduced into the system by purging the cell. Figure 7 shows the variation in the potential when the working electrode is BiVO_4_ and only the reduction potential has been plotted as there was no deflection observed for the oxidation peak. Upon insertion of these two scavengers, Fig. 7a shows the data when the hole scavenger has been introduced and Fig. 7b is for the electron scavenger. It can be observed that the removal of the light-induced holes does change the reduction potential however not substantially, there is still a viable shift in the reduction potential when light is impinged. This therefore suggests that the excited holes that are created in our BiVO_4_ do not contribute substantially to the experiment. This is analogous to the concept that holes in a n-type semi-conductor are not relevant to the electrical properties of that semiconductor. As such, it is hypothesized that in our experiment perhaps these holes are buried or not mobile enough so that they are not contributing to the redox behavior of BiVO_4_. Whether they are present (as expected upon light irradiation) or quenched in the presence of Na_2_SO_3_ they are not playing a vital role. However, it is important to note that in the presence of O_2_, a complete suppression of the voltage shift upon irradiation is observed (whether light is impinged or not). This observation indicates that the charge carriers that are being generated in the presence of light are directly contributing to the faradaic process apart from augmenting the capacitive storage. BiVO_4_ being an n-type semiconductor, it appears as the excited electrons are predominant contributors to charge storage and if they are quenched, then the effect of light irradiation is suppressed. Holes, however, perhaps as they are not as mobile in an n-type semiconductor and are not being quenched as rapidly (by not reaching the electrolyte/semi-conductor interface) their effect is not as substantial.

**Figure 8:**
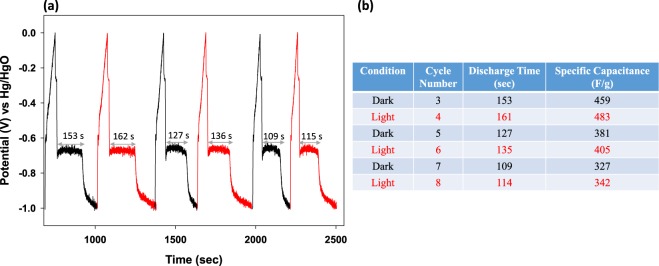
(**a**) Three charge-discharge cycles of BiVO_4_ rods acquired at 3 Ag^−1^ in the potential window 0.0 V to −1.0 V in dark and light. (**b**) Shows the discharge time and specific capacitance of BiVO_4_ in the presence and absence of light.

In order to gain further insight into whether the CV alterations actually had any effect in the charge-discharge behavior, Galvanostatic charge discharge measurements were performed at 3 A g^−1^ under the potential window 0.0 V to −1.0 V in the presence and absence of light, again in pulsed mode. Before initiating charge discharge measurements and so as to avoid any influence of the ‘activation process’^24^ first 20 CV cycles were carried out in dark for stabilization and subsequently, CD on the same working electrode was done. Shown in Fig. 8a is the CD curve whereby the same working electrode was irradiated with light for one CD cycle, and then kept in dark for the next CD cycle. This was alternated. It can be observed that there was a significant enhancement in the discharge duration on photoirradiating the working electrode, however the charging time was unperturbed. The enhancement in the overall discharge duration averaged over 3 sets of cycles in light and dark was *ca*. 30% on photoirradiating the working electrode with visible light. The absolute discharge time and specific capacitance for all the cycles in dark and in light have been tabulated, Fig. 8b. Also, on carefully observing the plateau in the presence of light, it was observed that the discharge plateau was more flat for a longer discharge duration and also, got shifted towards a positive potential and reverse behavior was observed for Co_3_O_4_ (Supplementary Fig. 9). Despite the degradation of BiVO_4_ and poor cycling stability, which could be circumvented on optimizing the electrolyte, we were able to augment the storage capacity of this material by simple illuminating the working electrode with visible light.

Confirmatory control experiments involving identical setup were also performed with other Bismuth based n-type semiconductors: Bi_2_MoO_6_ and Bi_2_WO_6_, and both of them showed similar behavior in the presence of light; reduction peak shifted towards positive potentials and there was an enhancement in the area under oxidation as well reduction peak. A shift in the reduction peak potential by *ca*. 10 mV and *ca*. 7 mV was observed for Bi_2_MoO_6_ and Bi_2_WO_6_, respectively under the potential window 0.0 V to −1.0 V at scan rate 20 mVs^−1^. Analogously the conventional battery material: LiCoO_2_ (an insulator)^28^ was also exposed to similar experimental conditions and the corresponding CV curve is shown in Supplementary Fig. 10. CV was acquired in the potential window 0.0 V to 0.55 V at a scan rate 20 mVs^−1^ (Supplementary Fig. 10a) and it can be observed that there is negligible deflection in the reduction peak potential in light-on or off conditions (Supplementary Fig. 10b). There is a lack of any change in the oxidation peak potential and in the peak area as well. Unlike other routes for integration where separate materials are combined into a heterogeneous electrode, this is a first ever report on bi-functional material where our work adapts electroactive semiconductors (BiVO_4_ or Co_3_O_4_) to play both the roles of light absorber and storage material.

## Conclusion

In summary, we have demonstrated that two different semiconductors *viz*. BiVO_4_ (n-type) and Co_3_O_4_ (p-type) having 3-D crystal structures (unlike conventional layered battery materials) can absorb and store solar energy in a single device. On impinging these electroactive materials with visible light, an augmentation in the area under the cyclic voltammogram was observed and simultaneously depending on the majority charge carriers, either of the faradaic processes (reduction in BiVO_4_ or oxidation in Co_3_O_4_) was facilitated. In case of BiVO_4_ on photo-illumination, reduction peak shifted towards positive potentials making the system thermodynamically more favorable, however, charging happened at the same potential. On the other hand, on impinging Co_3_O_4_ with visible light the charging potential was lowered (shift in the oxidation peak towards negative potentials) and the reduction peak potential remained unaffected. On introducing scavengers into the system, these perturbations in the CV could be suppressed indicating the direct influence of excited charge carriers on the faradaic behavior. In galvanostatic charge discharge measurements an enhancement was observed in the discharge plateau in presence of light. This is an alternate approach for coupling energy capture and storage where the main focus has been to minimize interfaces which could subsequently lead to better performance and charge transport efficiency.

## Methods

### Materials

Bismuth nitrate pentahydrate (≥99.99%), ammonium metavanadate (≥99.99%) and ammonium fluoride (≥99.99%) were purchased from Sigma Aldrich. Urea (99.0%) was procured from Fischer Scientific, while cobalt nitrate (≥99.0%), polyvinylidene fluoride, N-Methyl-2-Pyrrolidone and activated carbon powder (AR grade) were purchased from Himedia (India), and sodium oleate was purchased from TCI chemicals. All the chemicals were used without any further purification.

#### Synthesis of BiVO_4_ nanorods

Typically, 2 mmol (0.9701 g) Bismuth nitrate was dissolved in 36 ml of solvent (DI water and ethylene glycol with volume ratio 1:1) at room temperature. 5 g sodium oleate was added to the solution followed by stirring for 20 minutes. In 5 mL of water, 2 mmol (0.2338 g) ammonium metavanadate was dispersed and was added to the above solution. After stirring for 15 minutes, the solution was transferred into a stainless steel autoclave with a Teflon liner and heated at 180 °C for 24 hours. After cooling to room temperature, the reaction mixture was centrifuged and the pellet was washed with water, ethanol, and acetone. Finally the yellow product was dried under vacuum at 70 °C for 5 hours.

#### Synthesis of 1-D Co_3_O_4_

In a typical synthesis of Co_3_O_4_ precursor, 5 mmol (1.455 g) cobalt nitrate hexahydrate, 10 mmol (0.3704 g) ammonium fluoride, and 25 mmol (1.501 g) urea were dissolved in 50 ml deionised water under constant stirring for 10 minutes at room temperature. 15 ml of the precursor solution was transferred to 40 ml Teflon coated stainless steel autoclave at 120 °C for 24 hours. After cooling to room temperature, the pink cobalt precursor was washed thoroughly with deionized water and ethanol. Pink colored precursor was vacuum dried at 70 °C, and was then subjected to calcination in air at 400 °C for 4 hours to obtain pure spinel Co_3_O_4_.

#### Electrochemical Measurements

The electrochemical measurements were performed in a dry room at room temperature. Three electrode flat cell was employed with platinum foil as the counter electrode, Hg/HgO as the reference electrode, and 2 M NaOH as the electrolyte. The working electrode was prepared by mixing electroactive material (80 wt%), activated carbon (15 wt%) and polyvinylidene fluoride (5 wt%) with 1 mL of NMP to form a slurry that was dropcasted on graphite plate (area of coating, 1 cm^2^). Cyclic voltammetry (CV) and galvanostatic charge-discharge (CD) measurements were carried out using Bio-logic SP300 Galvanostat/Potentiostat Instruments.

### Characterisation

#### Brunauer-Emmett-Teller (BET) measurements

Specific surface area measurements were performed by nitrogen physisorption at 77 K on Micromeritics ASAP Surface Area and Porosity Analyzer. Prior to N_2_ physisorption, the samples were degassed at 150 °C for 12 hours.

#### X-ray diffraction

Powder X-ray diffraction (XRD) measurements were performed on PANalytical X’pertpro diffractometer equipped with monochromatic Cu Kα source (λ = 1.54056 Å) operating at 40 kV and 30 mA. The diffraction pattern was collected at room temperature with a 2θ angular range of 10° to 70° with a step size of 0.06°.

#### Electron microscopy

The elemental composition and surface morphologies of the samples were investigated by Field Emission Scanning Electron Microscopy (FE-SEM) on a Zeiss Ultra FEG 55 instrument at 5 kV operating voltage. HRTEM images were acquired on a FEI Tecnai-20 transmission electron microscope (TEM) equipped with a LaB_6_ filament operated at 200 kV.

#### Light Source

For photo illumination a 100 W Schott LED cold source lamp with a 400 nm long pass filter was used to obtain exclusive visible irradiation. The distance between the working electrode and the light source was 10 cm and was kept constant for all the experiments.

## Electronic supplementary material

Supplementary Information
