# Supplementary material for: Coupling Energy Capture and Storage – Endeavoring to make a solar battery
Source: Sci Rep. 2018 Aug 24;8:12752. doi: 10.1038/s41598-018-30728-8 (PMC6109126; doi:10.1038/s41598-018-30728-8)
Supplement: Supplementary file 1 — Supplementary Information [file 41598_2018_30728_MOESM1_ESM.docx]

**Coupling Energy Capture and Storage – Endeavoring to make a solar battery**

Yukti Arora^a^, Shateesh Battu^b^, Santosh Haram^b^, and Deepa Khushalani^a*^

^a^Department of Chemical Sciences, Tata Institute of Fundamental Research, Mumbai-400005, India

^b^Department of Chemistry, Savitribai Phule Pune University, Pune-411007, India

*email: [khushalani@tifr.res.in](mailto:khushalani@tifr.res.in)

**Fig. S1.** UV-Vis absorption spectra of BiVO_4_ and Co_3_O_4_ rods.

**Fig. S2.** CV of BiVO_4_ at higher scan rates.

**Fig. S3. (a)** and **(b)** represent the SEM images of the working electrode before and after 20 CV cycles. The SEM image (**a**) shows BiVO_4_ rods and also the flake like morphology which is the binder (PVDF) and activated carbon (AC) where as image **(b**) shows BiVO_4_ rods along with an ill-defined morphology which is now a combination of Bi_2_O_3_, NaVO_3_, V_2_O_7_ and the additives (PVDF+AC)**,** and (**c**) represents the X-ray diffraction pattern of the working electrode after 20 CV cycles and clearly shows the formation of Bi_2_O_3_ NaVO_3_, and V_2_O_7_.

**Fig. S4.** Representative diagram showing how the working electrode gets modified with the number of CV cycles; grey, orange and black are the working (graphite), reference (Hg/HgO) and counter (Pt foil) electrodes, respectively, assembled in a three electrode setup. Dark green block shows the electroactive material coated region onto the working electrode Yellow lines represent BiVO_4_ nanorods and yellow point stars represent the formation of Bi_2_O_3_, NaVO_3_ and V_2_O_7_. In dark during the first few cycles (b), we observe that electrode-electrolyte interaction increases and hence, the area under the CV curve increases but subsequently with number of cycles we observe a decrease in the area under the CV curve indicating that the concentration of BiVO_4_ rods is decreasing while that of the by products is increasing.

**Fig. S5.** Double layer capacitance measurements for determining electrochemically active surface area **(a)** represents cyclic voltammogram measured in a non-faradaic region of the voltammogram at different scan rates ranging from 20 to 400 mVs^-1^, and (**b**) represents the anodic current (capacitive) measured at -0.12 V vs Hg/HgO plotted as a function of scan rate.

**Fig. S6.** Representative diagram of the three electrode quartz flat cell showing BiVO_4_ coated working electrode being impinged with only visible component of light.

**Fig. S7.** Variation in the photoresponse which is the shift in the reduction peak potential of BiVO_4_ to different light intensities. The deflection in the reduction peak potential value increases with increase in the lamp power.

**Fig. S8. (a)** and **(b)** Variation in the area under oxidation and reduction peak, respectively, as a function of cycle number on irradiating Co_3_O_4_ rods with visible light, black curve is the baseline acquired in dark whereas red curve corresponds to three sets of cycles under ‘light on’ condition showing how the area under oxidation as well as reduction peak gets augmented. Data (Y-axes) in both the figures (a) and (b) is normalized with respect to area under the oxidation and reduction peaks in cycle number 10, respectively. Absolute values of enhancement in the areas under oxidation/ reduction peak vary because the potential window fixed to measure these area is different.

**Fig. S9.** Charge-discharge curves of Co_3_O_4_ rods acquired at 1 Ag^-1^ under the potential window 0.0 V to 0.55 V in the presence and absence of light.

**Fig. S10. (a)** CV curves of LiCoO_2_ in dark and light at 20 mVs^-1^ in 2 M NaOH, on photoirradiation there is lack of enhancement in the area under oxidation/ reduction peaks, and unlike BiVO_4_ or Co_3_O_4_, no shift in oxidation/ reduction peak is observed; **(b)** variation in the reduction peak potential (at 0.38 V) as a function of cycle number on pulsating with visible light. Data (Y-axis) has been normalised with respect to cycle number 2.
